# Amino acid metabolic reprogramming by gut microbiota: a novel metabolic checkpoint in the tumor microenvironment

**DOI:** 10.1080/19490976.2026.2719116

**Published:** 2026-08-18

**Authors:** Huiyu Lin, Xiya Deng, Yating Yang, Ruoyan Zhang, Yan-Dong Wang, Wei-Dong Chen

**Affiliations:** a Key Laboratory of Receptors-Mediated Gene Regulation and Drug Discovery, School of Basic Medical Science, Inner Mongolia Medical University, Hohhot, Inner Mongolia, People's Republic of China; b State Key Laboratory of Chemical Resource Engineering, College of Life Science and Technology, Beijing University of Chemical Technology, Beijing, People's Republic of China

**Keywords:** Amino acid metabolism, gut microbiota, cancer, metabolic reprogramming, tumorigenesis

## Abstract

The gut microbiota plays a crucial role in human health, significantly influencing various physiological functions such as amino acid metabolism, which is essential for maintaining homeostasis and preventing disease. Recent progress in gut microbiota research has highlighted the intricate relationships between gut microbial communities and host amino acid metabolism, particularly in the context of cancer. This review focuses on consolidating the latest advances regarding how gut microbiota–mediated amino acid metabolism contributes to cancer development and progression. While dysbiosis has been implicated in various diseases, such as metabolic disorders, autoimmune conditions, and neurodegenerative diseases, this review specifically examines the evidence linking gut microbiota and amino acid metabolism to cancer. Current studies suggest that alterations in gut microbial composition can lead to imbalances in amino acid availability and metabolism, thereby influencing systemic inflammation, oxidative stress, and immune responses that may promote tumorigenesis. Despite these insights, significant challenges remain, including identifying specific microbial strains, deciphering their metabolic pathways, and understanding their complex interactions with host factors in cancer progression. This review aims to summarize recent preclinical and clinical investigations elucidating the mechanisms linking the gut microbiota, amino acid metabolism, and cancer, and to evaluate potential therapeutic strategies targeting the gut microbiome to modulate amino acid metabolism and improve cancer treatment outcomes. By highlighting the gut microbiota as a promising target for innovative oncological therapies, this review provides insights into microbiome-based interventions targeting amino acid-related pathways in cancer.

## Introduction

The gut microbiota, which resides in the gastrointestinal tract, plays a variety of roles in maintaining human physiological function. Beyond its function in food breakdown and nutrient absorption, gut microbiota profoundly influences the complex interplay between amino acids and cancer. The intricate relationship between amino acid metabolism and gut microbiota is significant for understanding cancer pathogenesis and progression.^1^

The role of amino acid metabolic reprogramming has been increasingly recognized as important in the occurrence, development, and diagnosis of tumors. The primary function of amino acids is for protein synthesis in cells.^2^ They support metabolic processes crucial for the growth and survival of proliferating cells. Tumorigenesis and metastasis require upregulation of various metabolic pathways, including amino acid metabolic pathways, to meet their energy and nutrient needs. Evidence indicates that amino acids are closely linked to tumorigenesis and tumor immunity not only as nutrients but also as signaling molecules and epigenetic modulators.^3^

Emerging evidence suggests that the gut microbiota may modulate amino acid metabolism at immune checkpoints, thereby shaping immunosuppression and immunotherapy efficacy in cancer. For example, Du et al. reported that gut microbiota *Proteobacteria* may consume arginine, thereby increasing the suppressive capacity of Treg cells and inhibiting antitumor immune responses, thereby promoting tumor growth.^4^ Thus, elucidating the roles of the gut microbiota in amino acid metabolism can reveal how it contributes to metabolic reprogramming during tumor development, thereby identifying new potential targets for cancer treatment.

Here, we define the gut microbiota-mediated amino acid metabolic checkpoint as an extrinsic, upstream regulatory hub that shapes amino acid bioavailability. Unlike oncogene-driven intrinsic metabolic rewiring, this microbial checkpoint imposes substrate competition via balanced microbial and host enzymes to simultaneously regulate tumor proliferation, redox status, and anti-tumor immunity.

This review will concentrate on recent advances in understanding how gut microbiota‑mediated amino acid metabolism contributes to tumorigenesis. We propose a conceptual framework linking gut microbiota‑derived amino acid metabolites to three cancer hallmarks: immune evasion, metabolic competition, and ferroptosis resistance. We argue that microbial metabolism does not merely produce passive ‘signals’ but actively rewires host cellular machinery via specific receptors (e.g., the aryl hydrocarbon receptor (AhR)) and epigenetic cofactors (e.g., S‑adenosylmethionine, α‑ketoglutarate). Table 1 provides a pathway‑oriented overview integrating microbial species, metabolites, signaling pathways, tumor types, and their pro‑ or anti‑tumor effects. By synthesizing current evidence through this integrated framework, we aim to identify critical knowledge gaps and propose new directions for microbiota‑targeted or metabolite‑based cancer therapies.

**Table 1.** Amino acid metabolites in the gut microbiome context: Bidirectional functions in tumor development and immunotherapy.

Amino Acid	Microbe	Metabolite	Cancer Type	Effect of metabolite	Specific Mechanism	Ref.
Tryptophan	*L. johnsonii, C. sporogenes*	Indole-3-propionic acid (IPA)	Pan-cancer (melanoma, breast, colorectal)	Anti-tumor	Enhances CD8⁺ T cell stemness by increasing H3K27 acetylation at the Tcf7 super-enhancer, promoting Tpex differentiation and improving anti-PD-1 efficacy	[5]
	*L. reuteri*	Indole-3-acetic acid (IAA)	Colorectal Cancer, CRC	Anti-tumor	Activation of the pregnane X receptor (PXR) induces the production of interleukin-35 (IL-35) by immune cells	[6]
	*Acinetobacter radioresistens*	IAA	CRC	Anti-tumor	Inhibits intestinal stem cell turnover via AhR–Wnt-β–catenin axis, promoting homeostasis and protecting against tumorigenesis	[7]
	*Lactobacillus*	Indoles	Pancreatic Ductal Adenocarcinoma, PDAC	Pro-tumor	Drives tumor-associated macrophages (TAMs) toward a pro-tumor phenotype (AhR-dependent) and promotes cancer cell growth	[8]
	−	IAA	Hepatocellular Carcinoma, HCC	Anti-tumor	Protects against DEN-induced hepatocarcinogenesis by preserving catalase (CAT) and glutathione reductase (GR) activities and antioxidant gene expression, and by reducing DNA damage	[9]
	*Lactobacillus paracasei ZJUZ2-3*	IAA	Gastrointestinal Tumors	Anti-tumor	Acts as an AhR ligand to inhibit the MTDH/NF-κB axis, suppressing gastric cancer progression	[10]
	−	Indole-3-acetaldehyde (IALD)	CRC	Bidirectional (concentration-dependent)	At low concentrations (< 12.5 μmol/L) induces apoptosis and inhibits proliferation; at high concentrations (≥ 25 μmol/L) promotes EMT and invasion	[11]
	*Limosilactobacillus reuteri*	Indole-3-lactic acid (ILA)	HCC (post-TACE)	Alleviates post-TACE liver injury	Inhibits the ATPase activity of heat shock protein 90 (HSP90), thereby blocking the assembly and activation of the NLRP3 inflammasome, which suppresses macrophage-driven hepatic pro-inflammatory responses and alleviates post-TACE liver injury	[12]
	−	ILA	CRC	Anti-tumor	Directly blocks STAT3 phosphorylation sites, inhibiting HK2 pathway and glucose metabolism (AhR-independent), suppressing colorectal cancer growth	[13]
Tyrosine and Phenylalanine	−	*p*-Cresyl sulfate (PCS)	Bladder Carcinoma	Pro-tumor	Activates ROS/Src/FAK signaling to induce EMT, enhancing metastasis	[14]
	−	PCS	Clear Cell Renal Cell Carcinoma, ccRCC	Pro-tumor	Induces proliferation and migration via miR-21/HIF-1α axis	[15]
	−	PCS	Ovarian Cancer, OV	Pro-tumor	Promotes tumor growth and induces Cdh1/Bnip3-dependent mitophagy in Tim4 + tumor-associated macrophages, which reshapes antitumor immunity and ultimately aggravates cancer progression	[16]
	*Parvimonas micra*	*p*-cresol	Esophageal Squamous Cell Carcinoma, ESCC	Pro-tumor	Promotes PD‑L1 expression in CD4⁺ T cells and induces their differentiation into regulatory T cells (Tregs); simultaneously, the generated Treg cells suppress the antitumor function of CD8⁺ T cells through inhibitory cytokines such as IL‑10 and TGF‑β	[17]
Phenylalanine	−	Phenylacetate	Osteosarcoma	Anti-tumor	Induces cell cycle arrest and apoptosis in osteosarcoma cells	[18]
	−	Sodium phenylacetate	Breast Cancer	Anti-tumor	Blocks the downstream MEK/ERK signaling pathway by reducing c-Raf-1 protein levels and inducing its dephosphorylation, while Ras activation remains unchanged	[19]
	−	Phenylacetate	Prostate Cancer	Anti-tumor	Inhibits Skp2, preventing p27 ubiquitination, leading to p27 accumulation and cell cycle arrest	[20]
	−	Phenylacetylglutamine + Phenylacetic acid, 1:4	HCC	Anti-tumor	Arrests the cell cycle at the G0/G1 phase and induces apoptosis	[21]
Methionine	*Gut microbiota*	Hydrogen sulfide (H₂S)	Melanoma, CRC	Pro-tumor	Methionine restriction drives systemic sulfur depletion, inhibiting gut microbial hydrogen sulfide generation and attenuating both T-cell activation and anti-tumor immunity	[22]
	−	S-Adenosylmethionine (SAM)	Uveal Melanoma, UM	Anti-tumor (immune sensitizer)	Enhances anti-PD-1 efficacy by targeting EZH2 (epigenetic regulation)	[23]
	*Gut microbiota* (Dsr/Asr)	H₂S	CRC	Pro-tumor (association)	Hydrogen sulfide derived from microbial sulfur metabolism in the gut possesses genotoxic properties, and its overproduction is widely recognized as one of the potential drivers of colorectal carcinogenesis	[24]
Cysteine	Engineered bacterium	Cysteine depletion	PDAC	Anti-tumor	Disrupts antioxidant defenses in PDAC cells, enhancing ferroptosis (lipid ROS accumulation)	[25]
	−	Cysteine		Bidirectional regulation	In CD8⁺ T cells, cysteine-derived sulfur is partitioned into two pathways: NFS1-dependent iron-sulfur cluster synthesis to support proliferation, or glutathione (GSH) synthesis which dampens effector functions	[26]
	−	Cystine deprivation	Tumor microenvironment (CD8⁺ T cells)	Pro-tumor (immune suppression)	Cystine deprivation impairs glutathione synthesis; triggers CD36-mediated lipid accumulation and aberrant ROS production, resulting in dysfunction of CD8⁺ T cells, causing immunosuppression	[27]
	*Gut microbiota*	Arginine	CRC	Anti-tumor (combination therapy)	Targeting microbiota and arginase or supplementing L-arginine restores immune responses, enhances MEK inhibitor efficacy (upregulates MHC-I)	[28]
Arginine	*Gut microbiota*	Arginine	CRC	Pro-tumor	Massive degradation of L-arginine deprives CD8⁺ T cells of arginine, suppressing mTORC1 activity and abrogates their antitumor function; Concurrently, the breakdown products, including polyamines, facilitate DNA replication and cellular proliferation, which directly fuel the malignant transformation of intestinal epithelial cells	[29]
	*Streptococcus anginosus*	Ornithine	Gastric Cancer, GC	Pro-tumor	Promotes GC cell proliferation and metastasis while suppressing the differentiation and infiltration of CD8+ T cells	[30]
	−	Proline	PDAC	Pro-tumor	Collagen-derived proline serves as an alternative nutrient source to fuel the TCA cycle for survival	[31]
Proline	*Gut microbiota*	D-Proline	CRC	Anti-tumor	Caloric restriction alters microbiota, modulating D-proline and other metabolites to suppress colorectal cancer growth	[32]
Glutamine	*Gut microbiota*	Glutamine	Intrahepatic Cholangiocarcinoma, ICC	Pro-tumor	Inhibits ferroptosis in ICC cells by downregulating the ALK5/NOX1 axis, thereby promoting tumor growth both in vitro and in vivo	[33]

## The interplay of microbial-derived tryptophan metabolism and cancer pathogenesis

Tryptophan follows three main catabolic pathways in the gut: the indoleamine 2,3-dioxygenase 1-driven kynurenine route, the serotonin synthesis, and the gut microbiota-derived indole pathway.^34^ The first two pathways are host-driven, while only the third relies on gut microbiota. This review focuses exclusively on microbiota-mediated tryptophan metabolism.

Commensal genera, including *Bacteroides* and *Clostridium,* convert tryptophan into indole and its derivatives: indole-3-propionic acid (IPA), indole-3-acetic acid (IAA), indole-3-acetaldehyde (IALD), indoleacrylic acid (IA), and indole-3-lactic acid (ILA). Among these metabolites, *Clostridium sporogenes* generates most IPA through the reductive Stickland metabolism pathway.^35^ Minor IPA producers include *Peptostreptococcus asaccharolyticu*s, *Peptostreptococcus anaerobius*, and *Clostridium cadaveris*.

Multiple preclinical works confirm IPA improves αPD-1 immunotherapy across melanoma, breast and colorectal tumors. As a core mechanism, IPA remodels CD8⁺ T cell stemness by increasing H3K27 acetylation at the transcription factor 7 (*Tcf7*) super-enhancer, which generates progenitor-exhausted T_pex_ cells with sustained anti-tumor function.^5^ ^,^ ^36^ IPA exerts antitumor effects in preclinical models via multiple mechanisms, with causal support from gnotobiotic mouse experiments (e.g., C. sporogenes mono-colonization showing IPA-dependent AhR activation) and metabolite rescue studies (e.g., oral IPA reversing T-cell exhaustion).^5^ ^,^ ^37^

Gut commensals, including *Lactobacillus reuteri, Clostridium sporogenes*, *C. bartlettii*, and *Bifidobacterium spp*., convert tryptophan to IAA, which blocks precancerous colonic inflammation via IL-35, thereby preventing colitis-associated tumorigenesis.^6^ Intestinal *Acinetobacter radioresistens* secretes IAA to activate the AhR-Wnt-*β*-catenin axis, stabilizing intestinal stem cell homeostasis and suppressing tumor initiation.^7^ In liver cancer, IAA elevates the antioxidant activities of catalase and glutathione reductase to block Diethylnitrosamine-induced hepatocarcinogenesis.^9^ In gastric cancer models, IAA acts as an AhR agonist to suppress the oncogenic metadherin (MTDH)/nuclear factor kappa B (NF-κB) cascade and slow tumor progression.^10^ Contrasting protective effects appear in pancreatic ductal adenocarcinoma (PDAC). Hezaveh et al. demonstrated that IAA polarizes tumor-associated macrophages (TAMs) toward a pro-tumor phenotype via AhR activation, thereby accelerating PDAC cell proliferation.^8^ Overall, IAA exerts context-dependent dual effects on tumors. Its pro- or anti-tumor outcome is determined by intrinsic tumor features, including AhR expression, metabolic reliance and intracellular redox state.

Research on IALD remains limited, yet existing data reveal context-dependent biological activity. In colorectal cancer, IALD exhibits concentration-dependent dual functions: at low concentrations, it induces tumor cell apoptosis and inhibits proliferation, whereas at levels above 25 μmol/L, it drives epithelial-mesenchymal transition (EMT) and invasive phenotypes.^11^ Thus, in summary, IALD’s protective or pathogenic roles are dictated by its local concentration and tumor subtype.

In hepatocellular carcinoma (HCC), transarterial chemoembolization (TACE) operation disrupts commensal homeostasis and reduces *L. reuteri*-derived ILA. Restoring this metabolite or its producer relieves post-intervention liver injury and improves patient prognosis.^12^ For colorectal cancer (CRC), Ding et al. validated exogenous ILA blocks tumor proliferation both *in vitro* and *in vivo*. ILA directly binds STAT3 phosphorylation sites to suppress the Hexokinase 2-glucose metabolic axis, independent of AhR pathways.^13^ Though most anti-tumor evidence for ILA derives from CRC models, its immunomodulatory capacity suggests broader efficacy across solid tumors through dietary regimens rich in black rice or *β*-glucan to enrich ILA-producing commensals.^36^

**Figure 1.**
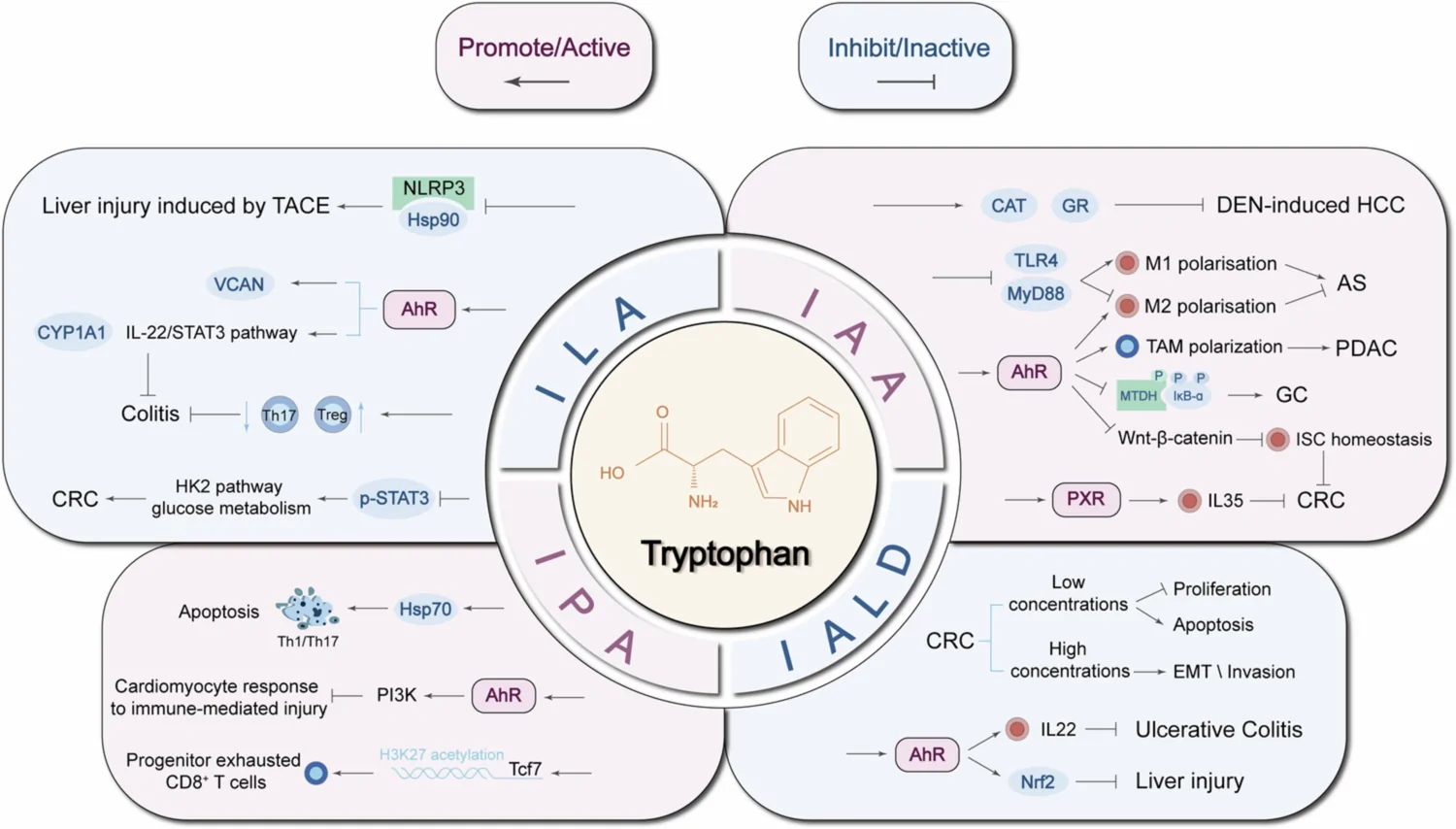
The dual and context-dependent roles of gut microbiota-derived tryptophan metabolites in cancer pathogenesis. Specific gut commensals convert dietary tryptophan into key indole derivatives, including indole-3-propionic acid (IPA), indole-3-acetic acid (IAA), indole-3-acetaldehyde (IALD), and indole-3-lactic acid (ILA). These metabolites engage multiple signaling pathways, particularly AhR, to exert dual, often opposing, effects across cancer types. Anti-tumor activities observed in colorectal cancer (CRC), gastric cancer (GC), and hepatocellular carcinoma (HCC) include enhanced anti-tumor immunity, suppression of NF-κB/STAT3 pathways, and reduced inflammation. In contrast, pro-tumor effects, observed in pancreatic ductal adenocarcinoma (PDAC) and under specific conditions, such as elevated IALD in CRC, are driven by the induction of an immunosuppressive tumor microenvironment. This intricate crosstalk highlights the potential of targeting microbial tryptophan metabolism as a novel therapeutic strategy for cancer. Note: Figure 1 was generated using Adobe Illustrator 2025.

Collectively, gut microbial tryptophan metabolites (IPA, IAA, IALD, ILA) engage AhR but yield divergent tumor outcomes, shaped by metabolite concentration, cancer subtype, and immune context (Table 1). IPA consistently strengthens anti-tumor immunity by sustaining CD8⁺ T cell stemness. IAA shows dual activity: it suppresses gastric and colorectal tumorigenesis yet promotes immunosuppressive TAM polarization in PDAC. ILA restrains CRC progression via STAT3 inhibition. These divergent phenotypes highlight a critical research gap: future work must map distinct microbial producers (e.g., *C. sporogenes* for IPA, *L. reuteri* for ILA/IAA) to their metabolite outputs, and clarify downstream immune and oncogenic signaling cascades across different cancer backgrounds (Figure 1).

**Figure 2.**
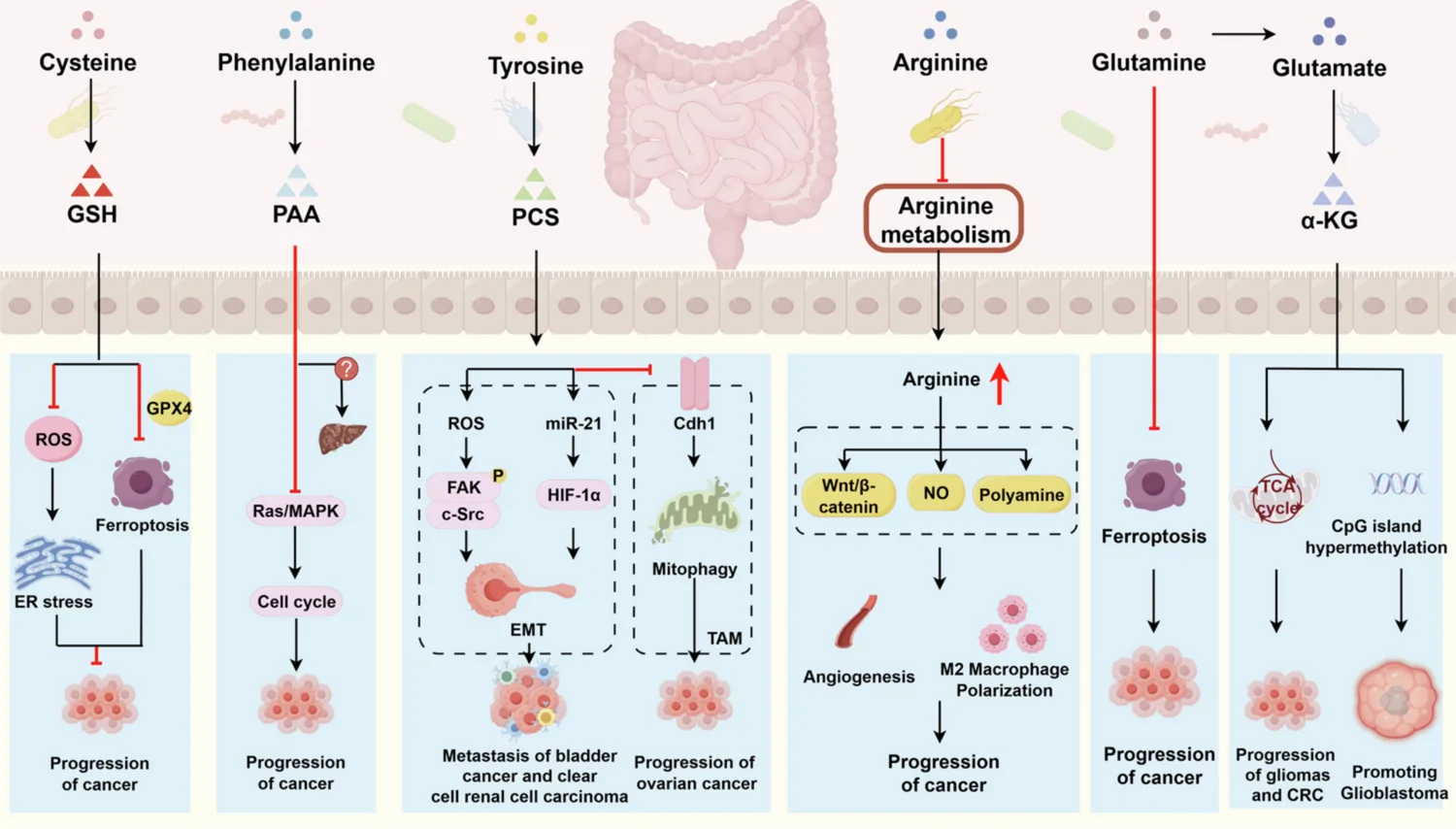
Regulatory network of gut microbiota-derived amino acid metabolites in cancer progression. The gut microbiota regulates cancer progression by critically reshaping intestinal amino acid availability through three core mechanisms: conversion of bioactive metabolites, competitive depletion, and enzymatic inhibition of catabolism. Diverse pathways exemplify this regulatory axis: PCS-induced EMT and metastasis in bladder/renal cancer; the anti-tumor effect of PAA via cell cycle arrest, though its functional relevance and mechanisms in hepatocellular carcinoma (HCC) require further clarification; GSH‑maintained cellular redox balance, leading to suppression of ROS accumulation, alleviation of ER stress, and resistance to ferroptosis; arginine-driven angiogenesis and M2 macrophage polarization and then promote tumor progression through NO/Wnt/β-catenin/polyamine signaling; and *α*-KG-promoted CRC and glioblastoma progression through modulation of the TCA cycle and CpG island hypermethylation. Note: Figure 2 was created by Figdraw.

**Figure 3.**
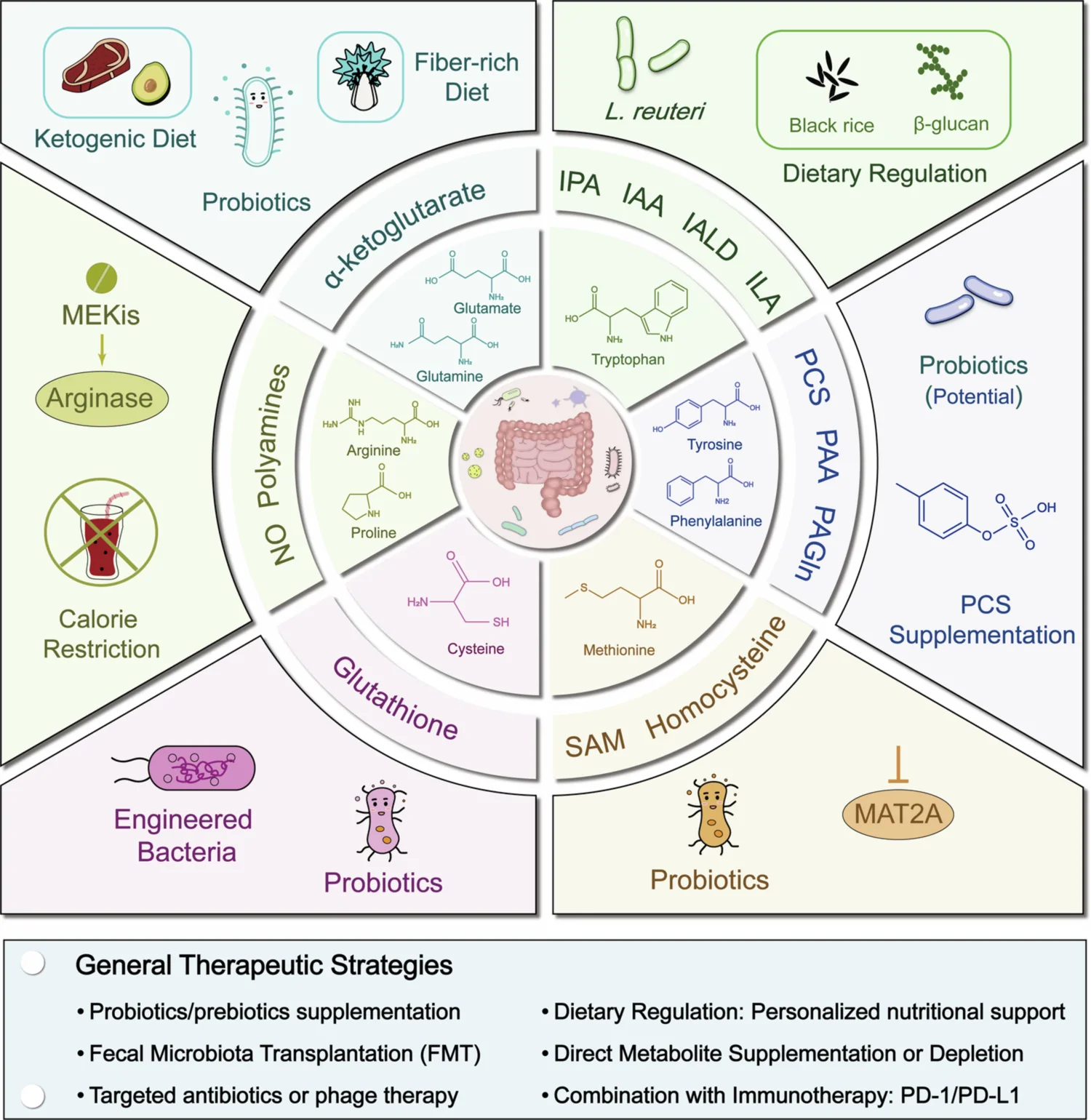
Multi-Layered Coordination of Gut Microbiota-Host Metabolic Crosstalk. This network is centered around the gut and its resident microbiota, encompassing various amino acid substrates (including tryptophan, phenylalanine, tyrosine, methionine, cysteine, arginine, proline, glutamine, and glutamic acid) and their derived key metabolites (e.g., IAA, PCS, SAM, glutathione, polyamines, and alpha-ketoglutarate). Modulation strategies targeting this metabolic network include dietary interventions (e.g., ketogenic diet, fiber-rich diet, black rice/β-glucan), metabolite supplementation (PAA supplementation), microbial interventions (e.g., probiotics like *Lactobacillus reuteri*, engineered bacteria), pharmacological/genetic targets (MEK inhibitors (MEKis), MAT2A), and lifestyle modulations (calorie restriction). These components and interventions work in concert to regulate host-microbiota metabolic homeostasis. Note: Figure 3 was generated using Adobe Illustrator 2025.

## Phenylalanine and tyrosine metabolism in cancer through gut microbiota

Beyond tryptophan, the metabolism of other aromatic amino acids, namely phenylalanine and tyrosine, is also significantly influenced by the gut microbiota with important implications for cancer. The metabolism of phenylalanine and tyrosine, two essential amino acids, is significantly influenced by the gut microbiota, which convert these amino acids into various metabolites, including phenylacetic acid (PAA) and *p*-cresyl sulfate (PCS) produced by *Clostridium* species.^38^ Elevated levels of PCS and PAA correlate with tumor-promoting inflammation and immune evasion. PCS is generated in the intestine when gut bacteria metabolize aromatic amino acids, including tyrosine and phenylalanine, into phenolic compounds.^39^ PCS exerts consistent tumor-promoting effects across multiple malignancies. In bladder cancer, PCS triggers EMT and metastatic spread through the ROS/Src/FAK cascade;^14^ in clear cell renal carcinoma, it facilitates cell proliferation and migration via miR-21/HIF-1α signaling;^15^ for ovarian cancer, PCS binds Cdh1 to boost Bnip3-dependent mitophagy in Tim4⁺ TAMs and accelerate tumor growth.^16^ Elevated circulating PCS has been correlated with colorectal, bladder, and ovarian tumors in human cohorts.^40^ However, current mechanistic evidence is limited to *in vitro* cell-based and xenograft models; rigorous *in vivo* verification, relying on germ-free animals or fecal microbiota transplantation (FMT) experiments, remains lacking. Consequently, the pro-tumorigenic role of PCS should be interpreted with considerable caution.

PAA and its derivative phenylacetylglutamine (PAGln) are synthesized from phenylalanine by the commensal microbial genes *porA* or *fldAIC* (*ppfor*).^41^ As early as 1986, Burzynski et al. reported that PAA and a mixture of phenylacetylglutamine and PAA in a 1:4 ratio, named antineoplaston AS2-1, exhibited interesting antineoplastic activity in breast carcinoma tissue culture and low acute and chronic toxicity in mice.^42^ Then, multiple reports show that the sodium salt of phenylacetic acid, phenylacetate, has anti-tumor activity. Samid et al. reported that phenylacetate is a promising therapeutic agent due to its dual properties: it not only promotes tumor cell maturation but also exhibits a favorable safety profile, being non-cytotoxic and non-carcinogenic.^43^ Park et al. reported that phenylacetate may be an effective chemotherapeutic agent for osteosarcoma through its growth-inhibitory actions, including cell-cycle arrest and apoptosis.^18^ Watanabe et al. demonstrated that sodium phenylacetate inhibits the Ras/MAPK signaling pathway, resulting in a reduction in c-Raf-1 protein levels in human and canine breast cancer cells.^19^ Shibahara et al. reported that phenylacetate suppresses Skp2, thereby inhibiting p27 ubiquitination, leading to p27 accumulation and cell cycle arrest in various prostate cancer cell lines.^20^ In 1996, Tsuda et al. reported that a 1:4 mixture of phenylacetylglutamine and PAA exhibited antitumor activity in HCC.^21^ Hershberger et al. reported that PAA was associated with HCC.^44^ PAA was significantly higher in plasma from those with HCC than in healthy individuals with cirrhosis. Thus, the function of PAA remains unclear and requires further investigation.

Gut microbiota, such as *Clostridium*, convert dietary phenylalanine and tyrosine into PCS and PAA. PCS drives pro-tumorigenic effects in bladder, renal, and ovarian cancers via ROS/EMT and HIF-1α/mitophagy pathways, while PAA exhibits anti-tumor activity (cell cycle arrest, differentiation) in osteosarcoma, breast, and prostate cancers, though its role in hepatocellular carcinoma remains unclear. This dichotomy illustrates how structurally similar microbial metabolites can exert opposing effects on tumor biology, depending on the target tissue and metabolic context.

## Methionine metabolism and gut microbiota in cancer

Shifting from aromatic amino acids, the sulfur-containing amino acid methionine also plays a critical role in the microbiota-cancer axis, particularly through its involvement in methylation processes. In 2023, Ji et al. demonstrated that dietary methionine restriction (DMR) promotes intestinal tumor progression by altering the gut microbiota, which in turn suppresses systemic anti-tumor T cell immunity.^22^ Methionine restriction consistently depleted the beneficial mucin-degrader *Akkermansia muciniphila* and increased *Bifidobacterium* in mice. In tumor-prone models, it also increased the *Firmicutes*/*Bacteroidetes* ratio and reduced colonic mucin production. Thus, they concluded that the gut microbiota is a crucial mediator through which dietary methionine restriction impairs host immunity and accelerates intestinal tumorigenesis.^22^ Dietary methionine modulates both the composition of the gut microbiota and its metabolic activity, leading to the production of precursors essential for the synthesis of S-adenosylmethionine (SAM). In hosts, a deficiency in external methionine, such as that caused by the inability of *Escherichia coli* to provide it, was found to impair the synthesis of both SAM and phosphatidylcholine.^45^ The findings of Wu et al. indicate that the gut microbiota plays a key role in regulating host methionine homeostasis. Dysregulation of this microbial community disrupts methionine balance, leading to local accumulation in the gut and elevated systemic concentrations. They proposed that the gut microbiota is a promising target for the treatment of diseases related to methionine metabolism.^46^

Cancer stem cells, critical for tumor initiation, rely on methionine-derived SAM to activate oncogenes via epigenetic regulation (e.g., DNA/histone methylation). Depleting methionine or inhibiting its conversion to SAM (e.g., by targeting MAT2A) suppresses tumor growth by disrupting methyl-donor supply.^47^ SAM has also been recognized as an enhancer of anti-PD-1 efficacy in uveal melanoma.^23^

SAM is the direct product of methionine activation by methionine adenosyltransferase (MAT), serving as the universal methyl donor. At the same time, homocysteine is derived indirectly from SAM via hydrolysis of S-adenosylhomocysteine (SAH) after donation of a methyl group. Methionine is metabolized to homocysteine, which can be remethylated to methionine or transsulfurated to cysteine.^48^ The cycling of homocysteine in methionine metabolism is intricately influenced by gut microbiota, which plays a critical role in methylation reactions and redox balance relevant to cancer progression.^49^ This metabolic pathway is crucial for maintaining cellular methylation processes, which are essential for DNA synthesis and repair, as well as regulating gene expression.^50^ Dysregulation of this cycle, particularly through elevated levels of homocysteine, has been associated with an increased risk of various cancers, including colorectal and breast cancer.^51^ Emerging evidence suggests that gut microbiota can modulate plasma homocysteine levels through multiple mechanisms, including the production of metabolites that either promote or inhibit homocysteine metabolism.^52^ For instance, certain gut bacteria can produce enzymes that facilitate the conversion of homocysteine back to methionine, potentially lowering plasma homocysteine levels and mitigating associated risks.^46^

Hyperhomocysteinemia, characterized by elevated levels of homocysteine in the blood, has been correlated with increased cancer risk. This condition can arise from genetic factors, dietary deficiencies (notably of B vitamins), and alterations in gut microbiota composition.^51^ ^,^ ^53^ Studies have demonstrated that specific microbial populations can influence homocysteine metabolism, thereby impacting systemic inflammation and tumorigenesis. For example, modulation of the gut microbiota through dietary interventions or probiotics has been shown to regulate homocysteine levels, which may influence cancer risk.^52^ Furthermore, the relationship between homocysteine and systemic inflammation suggests that elevated homocysteine levels may contribute to a pro-inflammatory state, a known facilitator of cancer progression.^54^ Therefore, targeting gut microbiota to regulate homocysteine levels presents a promising avenue for cancer prevention and treatment.

Gut microbial methionine metabolism directly affects the one-carbon pool and SAM availability, linking microbial activity to DNA/histone methylation and epigenetic regulation in cancer. While DMR promotes intestinal tumorigenesis by depleting beneficial *Akkermansia muciniphila*, SAM itself enhances anti-PD1 efficacy in uveal melanoma. Moreover, the gut microbiota regulates homocysteine recycling, and hyperhomocysteinemia is associated with increased cancer risk. Therefore, microbial modulation of the methionine-SAM axis represents a promising epigenetic checkpoint for cancer therapy.^55^ Methionine is a double‑edged sword: its availability sustains tumor growth and immunity, yet its restriction can paradoxically accelerate progression via microbiota‑mediated immune impairment. These opposing outcomes underscore that the net effect of methionine modulation in cancer therapy is highly dependent on tumor type, microbial composition, immune status, and the specific metabolic context.

The causal role of the gut microbiota in mediating the effects of methionine restriction has been convincingly demonstrated by Ji et al. using FMT and antibiotic-depletion experiments in mice.^22^ However, the translational relevance to human cancer remains associative, as most human data are based on dietary recall and plasma metabolite correlations.^56^ ^,^ ^57^

## Cysteine metabolism and gut microbiota in cancer

Cysteine, another key sulfur-containing amino acid, shares a close metabolic relationship with methionine and is similarly modulated by the gut microbiota to influence the tumor microenvironment. Cysteine is a conditionally essential amino acid. Cysteine enrichment in the gut lumen originates from two primary sources: the bacterial digestion of dietary proteins in the colon and the degradation of host-derived mucus proteins, such as cysteine-rich MUC2. The microbat-mediated utilization and metabolism of released cysteine significantly shape the gut microenvironment, contributing to host-microbiota crosstalk implicated in cancer development.^58^

In the gut microbiota equilibrium, cysteine serves as a key nutrient that acts not only as a metabolic player but also as a regulator, controlling pathogenic species such as *Clostridium difficile*, whose overgrowth can result from disruption of the normal flora. For *C. difficile*, cysteine functions both as a growth controller and as an inhibitor of toxin synthesis.^58^ The degradation of cysteine produces hydrogen sulfide (H₂S), which promotes the growth of certain microbes and provides protection against oxidative stress, particularly after antibiotic treatment. Cysteine-metabolizing bacteria are associated with oral health issues, such as abscesses and bad breath, and have been consistently linked to CRC.^24^ Although *Fusobacterium nucleatum* is a major focus in CRC research due to its strong association with tumors and patient prognosis, it is essential to note that it is sulfidogenic and one of many gut bacteria capable of generating H₂S from cysteine, a point often overlooked in studies.^24^

Microbiota and cancer cells exhibit a reciprocal relationship through cysteine metabolism. First, cancer cells can utilize cysteine produced by the microbiota as a direct metabolic fuel. Second, microbiota-derived cysteine products, such as glutathione (GSH) and H₂S, serve as antioxidants and key regulators of tumor energy metabolism.^59^ Beyond being a metabolic currency, cysteine also acts as a crucial regulator within the cancer metabolic network. Its versatile role is central to the metabolic reprogramming that allows cancer cells to survive the stressful tumor microenvironment and systemic imbalances in advanced disease. Additionally, alterations in cysteine metabolism by gut bacteria can alter the redox state of cancer cells, thereby influencing their proliferation and survival. For example, engineered bacteria that deplete cysteine in PDAC cells have been shown to enhance ferroptosis, a form of regulated cell death characterized by iron-dependent lipid peroxidation, by disrupting the antioxidant defenses of cells.^25^

Modulation of cysteine levels by the gut microbiota can also affect the efficacy of cancer therapies. For example, the presence of certain beneficial bacteria in the gut may enhance cysteine bioavailability, thereby supporting glutathione synthesis and protecting cancer cells from chemotherapy-induced oxidative stress. Conversely, microbial imbalance, characterized by the loss of beneficial bacteria and the overgrowth of pathobionts, can reduce cysteine availability and lead to glutathione depletion, making cancer cells more susceptible to therapeutic agents.^60^ ^,^ ^61^ This relationship underscores the importance of maintaining a healthy gut microbiota to achieve optimal cancer treatment outcomes, as it can influence both the metabolic landscape of tumors and the systemic response to therapy.

Within immune cells, especially CD8⁺ cytotoxic T cells, the primary effectors of anti-tumor immunity, cysteine availability dictates critical cellular processes: it fuels GSH biosynthesis to maintain redox homeostasis and effector function, and supports proliferation and metabolic fitness.^26^ Tumors frequently compete for cysteine, thereby inducing T cell exhaustion and dysfunction by limiting cysteine supply in the tumor microenvironment (TME).^27^ Conversely, commensal microbes, including *Bifidobacterium*, can elevate systemic cysteine levels, enhance GSH production in CD8⁺ T cells, and preserve their redox balance and anti-tumor activity.^26^ Gut microbial catabolism of cysteine also generates H₂S, which exerts context-dependent immune effects: physiological H₂S supports T-cell survival, whereas excessive H₂S from pathobiont-enriched bacteria promotes inflammation and immune suppression.^62^ This microbiota-cysteine-immune axis further modulates responsiveness to immune checkpoint inhibitors (ICIs), as restoring cysteine availability can reinvigorate T-cell function and sensitize tumors to immunotherapy.^26^ ^,^ ^27^ Collectively, cysteine serves as a pivotal mediator of metabolic crosstalk between the gut microbiota and anti-tumor immunity, representing a promising target for combinatorial cancer therapies.

Cysteine metabolism by sulfidogenic bacteria (e.g., *Fusobacterium nucleatum*) generates H₂S and GSH, which play dual roles: protecting cancer cells from ferroptosis (pro-tumor) while preserving CD8⁺ T cell redox balance (anti-tumor immune). The net outcome depends on the relative competition between tumor and immune cells for cysteine. Engineered bacteria that deplete cysteine in pancreatic cancer potentiate ferroptosis, whereas *Bifidobacterium* spp. enhance systemic cysteine to boost T-cell immunity. Thus, cysteine serves as a critical metabolic checkpoint linking the microbiota, ferroptosis, and immunotherapy response. While associative studies link cysteine-metabolizing bacteria (e.g., *F. nucleatum*) to CRC,^63^ direct causal evidence from germ-free or specific pathogen-free models is scarce. The engineered-bacteria study by Qiao et al. provides strong causal support in PDAC xenografts, but this is limited to a single preclinical model.^25^

## Arginine and proline metabolism and gut microbiota in cancer

The gut microbiota is implicated in modulating arginine and proline metabolism, which are critical pathways involved in various physiological processes, including nitric oxide (NO) production and polyamine synthesis.^64^ These metabolites are crucial for tumor growth and immune modulation, thereby influencing cancer progression. Studies have shown that alterations in the gut microbiota can affect arginine and proline levels, thereby influencing the tumor microenvironment and contributing to conditions such as cancer cachexia.^65^ Zhang et al. reported that cobimetinib, a MEK inhibitor, compromised antitumor immunity by altering the gut microbiota and reducing L-arginine levels via arginase-mediated production. Arginase inhibition or L-arginine supplementation restored immune responses.^28^ Xu et al. revealed that reduced arginine catabolism by the gut microbiota (specifically via the *E. coli* AST pathway) elevated arginine levels in the tumor microenvironment in colorectal cancer, activating oncogenic NO, polyamine, and Wnt/β-catenin pathways to drive angiogenesis, M2 polarization, and accelerated cancer progression.^29^ Streptococcus anginosus regulates the proliferation, migration, and invasion of tumor cells while also inhibiting the differentiation and infiltration of CD8 + T lymphocytes in the tumor immune microenvironment by altering the arginine metabolic pathway, ultimately promoting the onset and progression of gastric cancer.^30^ This suggests that microbiota play a crucial role in arginine metabolism in gastric cancer. Arginine, as a semiessential amino acid, regulates the tumor immune microenvironment, impacting cancer/immune cell proliferation, survival, and protein synthesis.

Proline, an amino acid released under cellular stress, exhibits a dual nature: its metabolism produces both ATP and ROS, which can lead to apoptosis or autophagy, yet it consistently acts as a pro-survival factor for tumor cells.^31^ Altered gut microbiota in patients with adenomas may contribute to changes in amino acid metabolism. Specifically, *Faecalitalea* and *Butyricimonas* were enriched before polypectomy, normalized after removal, and showed a positive correlation with adenoma-specific proline and serine levels. This correlation implies microbiota changes could either drive or be driven by amino acid alterations.^66^ The gut microbiota can influence proline availability through fermentation, which can either enhance or inhibit its production depending on the microbial composition.^65^ The report by Zhang et al. indicated that calorie restriction alters the abundance of gut microbes (e.g., *the Nitrospirae phylum and the genus Lactobacillus)* in a mouse model of colorectal cancer, thereby modifying metabolites such as D-proline and isoleucyl-valine, which contribute to the suppression of CRC growth.^32^ Integrated microbiome-metabolomics analysis in the report by Chen et al. indicated that gut microbiota regulation suppresses MC38 xenograft tumor growth, potentially through disruption of host lipid metabolism (sphingolipids, glycerophospholipids, primary bile acids) and amino acid metabolism (e.g., arginine-proline).^65^

Thus, the gut microbiota not only affects the availability of these critical amino acids but also modulates the metabolic pathways that utilize them, impacting tumor biology and the overall health of the host. In summary, the gut microbiota modulates arginine/proline metabolism through both depletion and polyamine production, with context-dependent effects on tumor immunity and progression. Targeting this axis, whether through arginase inhibition, polyamine blockade, or microbial editing, holds therapeutic promise, though rigorous mechanistic and clinical validation remains essential.

Gut microbiota regulate arginine availability through arginase production (e.g., *Proteobacteria*) or the AST pathway (e.g., *E. coli*), thereby controlling NO, polyamine, and Wnt/β catenin signaling. Arginine depletion generally suppresses anti-tumor immunity (Treg activation, CD8⁺ dysfunction), while excess arginine drives angiogenesis and M2 polarization. Proline, released from collagen under stress, supports pancreatic cancer cell survival. Together, the arginine-proline axis exemplifies how microbial competition for semi-essential amino acids reshapes the tumor metabolic landscape and immune evasion.

## Glutamine and glutamate metabolism and gut microbiota in cancer

Glutamine fuels tumor growth by entering the tricarboxylic acid (TCA) cycle as *α*-ketoglutarate, thereby providing ATP, maintaining redox balance, and serving as a biosynthetic precursor.^67^ Cancer cells exhibit high metabolic plasticity, enabling them to adapt their dependency on this pathway during metastasis to varying microenvironments.^68^ For instance, glutamine deprivation increases reliance on alternative nutrients such as aspartate, asparagine, branched-chain amino acids, or glucose.^69^ Conversely, prolonged inhibition of glycolysis can activate AMPK, upregulating glutamine metabolism and/or fatty acid utilization to support survival.^70^

The gut microbiota consumes glutamine for nitrogen and energy,^71^ thereby altering its availability for cancer cells. Changes in microbial composition exert significant, context-dependent effects on glutamine metabolism. For example, *Fusobacterium nucleatum* co-cultured with patient-derived CRC cells exerts protumorigenic effects through metabolic reprogramming, increasing both formate production and glutamine metabolism.^72^ This enhanced glutamine metabolism can also contribute to cancer treatment resistance.^73^ Emerging evidence indicates that the gut microbiota and its metabolites play a key role in the development of triple-negative breast cancer (TNBC). Liu et al. reported that four serum metabolites, L-glutamine, citrate, creatinine, and creatine, along with nine fecal metabolites, were associated with microbiota changes in the TNBC group. The negative correlation between *Anaerococcus* and L-glutamine suggests that these bacteria either inhibit glutamine utilization or actively regulate glutamine metabolism through specific mechanisms in the TNBC environment.^74^ Zhang et al. reported that the gut microbiota altered glutamine metabolism to inhibit ferroptosis in intrahepatic cholangiocarcinoma by regulating the activin receptor-like kinase 5 (ALK5)/NADPH oxidase 1 (NOX1) axis.^33^

Alterations in the gut microbiota are also linked to brain tumors, including glioma and glioblastoma, with glutamine serving as a key mediator of this association. Specifically, glioma cells are highly dependent on glutamine, which serves as a critical substrate for both energy metabolism and nitrogen supply, two fundamental processes that sustain tumor growth and proliferation. This excessive glutamine demand by glioma cells results in decreased glutamine levels in patients’ blood. Notably, blood-brain barrier (BBB) damage during tumor progression further facilitates the transport of available glutamine into the brain, allowing tumors to effectively hijack this essential nutrient supply.^75^ In addition to supporting tumor energy metabolism, this hijacked glutamine also contributes to the establishment of an immunosuppressive tumor microenvironment, which subsequently promotes glioma progression. Given the central role of glutamine in glioma development, therapeutic strategies targeting glutamine availability have been developed. These approaches, which involve modulating the gut microbiome via ketogenic diets, probiotics, prebiotics, and fiber-rich diets, are designed to limit tumor growth by restricting access to this key metabolic resource.^76^ Furthermore, the interaction between glutamine metabolism and the immune response within the tumor microenvironment is increasingly recognized as a critical determinant of glioma progression.

Tumor cells often compete with immune cells for glutamine, a nutrient that is also essential for T cell activation and proliferation. Because tumors utilize glutamine more efficiently than immune cells, they gain a competitive edge, leading to immune evasion and impairment of the anti-tumor immune response.^77^ ^,^ ^78^ Many omics associations cannot establish causality. In TNBC, Anaerococcus abundance negatively correlates with circulating glutamine, yet confounders such as diet and treatment obscure the direction of effects.^74^ By contrast, antibiotic depletion and glutamine rescue assays, together with isotope tracing, provide causal evidence that gut microbiota modulate glutamine metabolism to suppress ferroptosis in intrahepatic cholangiocarcinoma.^33^

Glutamate plays a complex, dual role in tumor development. On one hand, high levels of glutamate promote inflammation and induce obesity and diabetes, both of which are associated with early-onset colorectal cancer in animal models. On the other hand, glutamate and its metabolic pathways can also exert antitumor effects. In the report by Ding et al., a bioactive compound, Astragalus polysaccharides (APS), significantly regulated the growth of various microorganisms, including *Bifidobacterium pseudolongum*, *Lactobacillus johnsonii*, and *Lactobacillus*, leading to an increase in metabolites such as glutamate and creatine, which could potentially control tumor growth in melanoma mouse models.^79^ Spearman correlation analysis revealed a decrease in *B. pseudolongum* and an increase in *L. johnsonii*, both of which were associated with changes in creatine and L-glutamate. Notably, creatine and L-glutamate are associated with antitumor effects. Zhu et al. reported that the combination of *L. casei* and *L. reuteri* inhibited the proliferation, migration, and invasion of pancreatic cancer cells, as well as tumor growth in pancreatic cancer-bearing mice. Additionally, it reduced the abundance of *Alloprevotella* in the gut of pancreatic cancer-bearing mice, while increasing fecal azelate and glutamate. They did not mention the role of glutamate in pancreatic cancer.^80^ Glutamate metabolism was also associated with gastric cancer.^81^ Together, these findings suggest that glutamate may exert context-dependent regulatory functions across different tumor types.

Gut bacteria regulate L-glutamate signaling in three ways: through their own L-glutamate metabolism, by breaking down dietary L-glutamate, and by producing L-glutamate. In CRC, microbiome analysis revealed increased breakdown of L-glutamate, accompanied by reduced production of both L-glutamate and L-glutamine, resulting in low L-glutamate levels in the colon.^82^ Cancer cells not only display an endogenous adaptive response to oxidative stress by increasing the expression of colon-specific antioxidant enzymes and molecules, but they also cross-talk with the tumor microbiota to decrease oxidative stress by degrading L-glutamate.^83^

Beyond CRC, the role of glutamate metabolism has also been explored in other cancer types, though with distinct mechanistic implications. In oral squamous cell carcinoma (OSCC), oral antibiotics accelerate progression by depleting gut microbiota, elevating tyrosine levels, and suppressing glutamate. Mechanistically, tyrosine promoted SCC7 proliferation, T-cell PD-1 expression, and necroptosis, while reduced glutamate impaired CD11c⁺ IL-10 production. Glutamate supplementation or fecal microbiota transplantation reversed tumor promotion by metabolic rebalancing, implicating antibiotic stewardship in OSCC management.^84^ At the epigenetic level, glutamate metabolism intersects with key regulatory pathways in cancer. Glutamate-derived alpha-ketoglutarate (*α*-KG) serves as a critical cofactor for DNA and histone demethylases, linking glutamate metabolism to epigenetic regulation. Disruption of the glutamate, *α*-KG equilibrium, notably through IDH1/2 mutations that convert *α*-KG to D-2-hydroxyglutarate (D2HG), inhibits *α*-KG-dependent demethylases. This blockade induces CpG island hypermethylation, silencing tumor suppressor genes and directly promoting glioblastoma pathogenesis.^76^

The gut microbiota-glutamate axis plays a dual role in cancer progression and immune evasion, acting as both a driver of tumor malignancy and a potential target for metabolic immunotherapy. On the one hand, tumor cells actively reprogram glutamate metabolism to support their growth. For instance, in gliomas and CRC, glutamate is converted to *α*-KG to replenish the tricarboxylic acid (TCA) cycle, thereby fueling energy production and biosynthetic demands.^82^ Moreover, tumor-secreted glutamate, mediated by the xCT transporter, creates an immunosuppressive microenvironment by inhibiting T‑cell proliferation through the metabotropic glutamate receptor mGlu5R, thereby promoting immune escape.^85^ On the other hand, microbial imbalance can exacerbate these processes. For example, cadmium exposure in breast cancer models elevates gut glutamine levels (a glutamate precursor) by suppressing glutamine‑consuming bacteria such as *Blautia obeum* and *Clostridium butyricum*, thereby accelerating tumor growth.^86^ Conversely, modulating the gut microbiota through probiotics, prebiotics, or ketogenic diets reduces the availability of glutamine and glutamate, thereby inhibiting tumor proliferation.^76^ ^,^ ^87^ ^,^ ^88^ Taken together, the gut microbiota-glutamate axis represents a critical interface linking cancer pathogenesis to metabolic immunotherapeutic strategies.^76^

Glutamine fuels the TCA cycle via *α*-KG and supports glutathione synthesis, positioning it as a key metabolic and immune node in cancer. Gut microbiota, such as *Fusobacterium nucleatum*, enhance glutamine utilization, thereby promoting chemoresistance. Conversely, *Paraburkholderia fungorum* inhibits cholangiocarcinoma through glutamate pathway modulation. Glutamate-derived *α*-KG also serves as a cofactor for DNA/histone demethylases, linking microbial glutamate metabolism to epigenetic reprogramming (e.g., *IDH* mutations in glioma). Thus, the microbiota-glutamine/glutamate axis integrates energy metabolism, redox balance, and epigenetics in cancer (Figure 2).

In summary, the metabolism of glutamine and glutamate is intricately linked to cancer progression, and this link is critically shaped by the gut microbiota. Glutamate exerts dual, context-dependent effects: it promotes malignancy via the TCA cycle, inflammation, and xCT/mGlu5R-mediated T-cell suppression, yet can also exert antitumor effects through microbiota-mediated production, *α*-KG-dependent epigenetic regulation (in IDH wild-type tumors), and reversal of immunosuppression. The net outcome is dictated by tumor type, metabolic context, and gut microbial composition. Thus, these amino acids play a dual role: they act as essential substrates fueling tumor metabolism while also serving as modulators of the immune response. This dual functionality highlights their potential as therapeutic targets. Future research should therefore focus on elucidating the specific mechanisms by which microbial glutamine/glutamate metabolism influences tumor behavior and the immune landscape, paving the way for targeted therapeutic strategies.

## Intratumoral microbiota, amino acid metabolism, and tumor progression

Accumulating evidence across cancer types has established the intratumoral microbiota as a distinct regulator of local amino acid metabolism, influencing tumor progression, immune evasion, and therapy response. In intrahepatic cholangiocarcinoma (ICC), Chai et al. demonstrated that *Paraburkholderia fungorum*, enriched in paracancerous tissues and negatively correlated with CA199 levels, exerts antitumor activity by modulating alanine, aspartate, and glutamate metabolism, as confirmed by integrated metabolomic and transcriptomic analyzes.^89^ Conversely, in esophageal squamous cell carcinoma (ESCC), Cheng et al. identified *Parvimonas micra* as a tumor-enriched pathobiont that secretes *p*-cresol, a tyrosine fermentation product, o elevate ROS in CD4⁺ T cells, thereby inducing FOXP3⁺ regulatory T cell (Treg) differentiation and creating an immunosuppressive microenvironment that accelerates tumor growth.^17^ In pancreatic cancer (PC), Luo et al. revealed that *Pseudomonas* dominates the intratumoral microbial community and is significantly associated with altered amino acid metabolic pathways, particularly glutamine/glutamate and branched-chain amino acid metabolism, with high *Pseudomonas* abundance correlating with poor patient survival; however, the causal relationship remains to be experimentally validated.^90^ In gastric cancer, intratumoral *Streptococcus anginosus* consumes arginine via arginine deiminase (ADI), depleting TME of arginine and promoting tumor proliferation while suppressing CD8⁺ T cell immunity.^30^ In CRC, intratumoral *Fusobacterium nucleatum* converts arginine into ornithine and downstream polyamines (e.g., putrescine), thereby activating Wnt/β‑catenin signaling and driving malignant proliferation.^91^

Thus, intratumoral microbiota shape amino acid metabolism in a cancer‑type‑specific manner, either depleting arginine to impair T cell function or supplying polyamines to fuel tumor growth and immunosuppression, highlighting the microbiota‑arginine axis as a promising therapeutic target. Collectively, these studies converge on a paradigm in which intratumoral microbiota, ranging from antitumor *Paraburkholderia* to protumor *Parvimonas*, *Pseudomonas*, and oral-derived taxa, reshape the local amino acid landscape (tryptophan, tyrosine, glutamine, arginine, and branched-chain amino acids) to orchestrate cancer cell metabolism, immune polarization, and treatment sensitivity. However, a recurring limitation across these reports is the predominance of correlational multi-omics data over mechanistic causality; future studies employing germ-free models, bacterial ablation, and stable isotope tracing are urgently needed to firmly establish causal links and translate these microbial-metabolic axes into actionable therapeutic targets.

## Challenges and future directions

The gut microbiota cannot be overlooked when developing diet-based anticancer strategies. Anticancer treatments-radiotherapy, chemotherapy, and immunotherapy-dynamically reshape the gut microbiota, which in turn reciprocally modulates therapeutic responses through its compositional and functional properties. The gut microbiota has emerged as a key player in the complex interplay between dietary amino acids and cancer. Microbiota-derived amino acid metabolites can drive cancer progression and influence treatment efficacy. Therefore, understanding the intricate relationship between amino acid metabolism and the gut microbiota is essential for deciphering cancer pathogenesis and progression. Recent studies have highlighted that the gut microbiota modulates the metabolism of various amino acids, generating metabolites that either promote or inhibit cancer development.

Longitudinal studies are crucial for capturing dynamic changes in microbiota composition and function over time, especially during different stages of cancer development and treatment. Furthermore, the role of the gut microbiota in modulating systemic metabolism has implications for cancer cachexia, a condition marked by severe weight loss and muscle wasting, underscoring the need for targeted interventions to improve supportive oncology care. Recent findings suggest that specific gut microbiota profiles may influence the severity of cachexia and overall metabolic health in cancer patients, pointing to potential avenues for therapeutic modulation.

The intricate interplay between gut microbiota, amino acid metabolism, and cancer poses a multifaceted challenge that requires a comprehensive approach to uncover the underlying mechanisms. Given the complexity of host, microbiota interactions, particularly in amino acid metabolism, integrating multi-omics technologies, such as genomics, transcriptomics, proteomics, and metabolomics, is essential. Such an integrative framework can yield valuable insights into the causal relationships and mechanisms governing these interactions, especially in the context of cancer progression and treatment response.

Personalized microbiota modulation strategies, including probiotics, prebiotics, and dietary interventions, hold promise for enhancing treatment efficacy and mitigating adverse effects in cancer patients. However, these strategies require rigorous clinical validation to establish their safety and effectiveness across diverse patient populations. The marked variability in individual gut microbiota compositions underscores the need for personalized approaches that account for genetic, environmental, and lifestyle factors influencing gut microbiota dynamics. Future research should focus on identifying specific microbial signatures associated with treatment outcomes and developing tailored interventions that optimize the therapeutic potential of microbiota modulation.

Furthermore, elucidating the roles of gut microbiota in cancer cachexia and systemic metabolism could lead to innovative supportive care strategies that address the metabolic derangements commonly observed in cancer patients. By clarifying the mechanisms by which the gut microbiota influence energy balance, muscle metabolism, and appetite regulation, researchers can identify novel intervention targets that may alleviate the burden of cachexia and improve patients' quality of life. Integrating microbiota research into clinical oncology practice represents a significant step toward enhancing patient outcomes and developing comprehensive care strategies that account for the multifactorial nature of cancer and its metabolic consequences.

Throughout this review, several themes recur: immune suppression, redox regulation, ferroptosis, and epigenetic remodeling. However, these are not independent; microbiota-derived amino acid metabolites act as central hubs. For instance, hydrogen sulfide derived from cysteine metabolism from *Fusobacterium* exerts multiple effects: it enhances redox regulation via glutathione synthesis, confers ferroptosis resistance by suppressing lipid-derived ROS, and drives immunosuppression through M2-like macrophage polarization. This convergence suggests a combinatorial perturbation mechanism whereby a single microbial metabolite coordinates multiple hallmarks of cancer. We propose that future therapeutic strategies should target the upstream microbiota-mediated enzymatic pathways rather than individual downstream effectors.

Despite the diversity of amino acid substrates and their microbial metabolites, several recurrent mechanistic motifs emerge. First, many microbiota-derived metabolites act as host receptor ligands, notably AhR for tryptophan indoles, as well as ROS signals from phenylalanine/tyrosine and NO from arginine. These shared signaling hubs, NF-κB, STAT3, and Wnt/β-catenin, converge to modulate tumor cell proliferation, angiogenesis, and immune cell polarization. Second, amino acid availability dictates immune cell metabolism and function, establishing a three-way competition among tumor, immune cells, and microbes. This competition is particularly evident for cysteine (GSH synthesis and T-cell redox fitness), arginine (polyamine and NO production), and glutamine (TCA cycle anaplerosis), where microbial consumption or production of these amino acids directly tips the balance between immune activation and exhaustion. Third, redox regulation and cell death modalities, apoptosis, ferroptosis, and autophagy, are jointly influenced by cysteine, methionine, and glutamine metabolism, coupling the gut microbial composition to tumor cell stress resilience. Finally, methionine-derived SAM and glutamine-derived *α*-KG serve as essential cofactors for epigenetic modifiers, linking microbial amino acid metabolism to DNA/histone methylation patterns that govern oncogene silencing and tumor suppressor expression. Collectively, these common themes suggest that the gut microbiota-amino acid-cancer axis operates not as a collection of parallel, independent pathways, but as an integrated network whose nodes are differentially weighted across tumor types and stages.

Critical translational barriers necessitate in-depth elaboration. First, profound interindividual heterogeneity in gut microbiota composition severely undermines the efficacy of universal microbial interventions, while inconsistencies in sequencing workflows and patient inclusion criteria across studies have led to poor reproducibility among clinical cohorts. Uncontrolled dietary composition further acts as a potent confounding variable, distorting correlations between microbial metabolites and cancer outcomes. On the analytical front, technical hurdles, including interference from complex biological matrices and the lack of standardized reference materials, impede accurate, uniform quantification of gut microbiota-derived amino acid metabolites.

Beyond these methodological challenges, several major clinical gaps remain unresolved regarding the feasibility and safety of microbiota-based therapies. Oral probiotics often exhibit unstable colonization efficiency in patients, and the long-term safety profile of such interventions in immunocompromised cancer populations has yet to be characterized. Moreover, no validated microbial or metabolite biomarkers currently exist to guide stratified patient selection. Notably, engineered bacteria remain confined to laboratory research and are not yet deployable in clinical practice. Likewise, the synergistic potential of microbial agents combined with immune checkpoint blockade cannot be generalized from isolated animal studies alone; rigorous validation in large-scale prospective human cohorts is essential.

Another challenge that currently limits clinical translation is the predominance of associative evidence. To move the field forward, future research must prioritize experimental designs that directly test causality. We advocate for the routine use of gnotobiotic models, FMT with defined consortia, genetic knockout of microbial metabolic genes, and stable-isotope-labeled substrate tracing *in vivo*. Moreover, metabolite rescue experiments, in which a candidate microbial metabolite is supplemented to phenocopy or reverse the effects of microbial depletion—are particularly powerful. Only through such rigorous causal frameworks can we confidently prioritize targets for clinical development.

To address these obstacles, future translational efforts should prioritize the following directions: First, establishment of standardized multi-omics detection pipelines; second, strict dietary control in clinical trial designs; third, application of machine learning algorithms to correct for interpatient microbiome variability; and dedicated phase I/II trials to evaluate the safety, optimal dosage, and timing of microbiota-based adjuvant therapies. Ultimately, rigorous clinical validation remains an indispensable prerequisite for the safe and effective integration of any of these strategies into routine oncology practice.

Collectively, this review establishes the unified paradigm of gut microbiota-mediated amino acid metabolic checkpoint: commensals act as an upstream regulatory switch governing systemic amino acid pools to jointly control tumor growth, redox balance and anti-tumor immunity. Instead of repeatedly recapitulating known metabolite phenotypes across distinct amino acid pathways, we highlight three core research priorities: standardizing gnotobiotic, isotope tracing and metabolite rescue experiments to distinguish causal relationships from correlational omics data; developing personalized microbial interventions matched to patient microbiome and tumor subtypes to resolve interindividual heterogeneity; and deploying longitudinal multi-omics to dissect gut-intratumoral metabolic crosstalk during cancer progression. Combinatorial strategies targeting microbial enzymes and host amino acid metabolism, alongside microbiota-based supportive care for cancer cachexia, represent promising translational directions, and multidisciplinary research integrating microbiology, tumor metabolism and clinical oncology will unlock novel microbiota-metabolite anti-cancer therapeutics centered on this metabolic checkpoint (Figure 3).

## Data Availability

Data sharing does not apply to this article as no new data were created or analyzed in this study.
